# Commentary: Evaluation of post-COVID mortality risk in cases classified as severe acute respiratory syndrome in Brazil: a longitudinal study for medium and long term

**DOI:** 10.3389/fmed.2025.1558264

**Published:** 2025-05-16

**Authors:** Rubens C. Costa-Filho, José Paulo G. Leite, Marco Aurélio Horta, Felipe Gomes Naveca, Felipe Saddy, Hugo C. Castro de Faria Neto

**Affiliations:** ^1^Laboratory of Immunopharmacology, IOC/FIOCRUZ, Rio de Janeiro, RJ, Brazil; ^2^Estácio Medical School - IDOMED, Rio de Janeiro, Brazil; ^3^Laboratory of Comparative and Environmental Virology, IOC/FIOCRUZ, Rio de Janeiro, RJ, Brazil; ^4^Biosafety Level 3 Platform (NB3), Rio de Janeiro, RJ, Brazil; ^5^Oswaldo Cruz Institute, Fiocruz, Rio de Janeiro, Brazil; ^6^Leônidas and Maria Deane Institute, Rio de Janeiro, Amazonas, Brazil; ^7^Intensive Care Unit, Hospital Pró-Cardíaco, Rio de Janeiro, RJ, Brazil; ^8^Faculdade de Medicina, Universidade Estácio de Sá, IDOMED, Rio de Janeiro, RJ, Brazil

**Keywords:** COVID-19 mortality analysis, observational study limitations, vaccine safety misinterpretation, cox regression model assumptions, epidemiological data quality

## Introduction

The study by Rodrigues and Andrade explores medium- and long-term mortality risks among individuals with severe acute respiratory syndrome (SARS) due to COVID-19 in Brazil, using data from the SIVEP-Gripe database. Their analysis, based on Cox proportional hazards models, suggests an increased mortality risk over time among vaccinated individuals. However, while addressing a critical public health issue, several methodological flaws undermine the reliability of these conclusions.

Key concerns include inappropriate use of the SIVEP-Gripe database, which was not designed for long-term mortality analysis, the exclusion of early deaths, which introduces survival bias, and insufficient statistical adjustments, particularly regarding proportional hazards assumptions. These issues limit the scientific rigor of the study and raise broader concerns about its potential to misinform public health discussions on vaccine safety and effectiveness.

A recent report by the Brazilian Ministry of Health (1) has explicitly criticized the misuse of the SIVEP-Gripe database for long-term mortality studies, warning that such methodological oversights may lead to misleading conclusions about COVID-19 and vaccination outcomes. If left unaddressed, these issues risk fueling misinformation and may inadvertently provide misleading narratives that undermine public confidence in vaccines. This commentary critically examines these limitations, presenting a structured analysis of the study's methodological concerns and their potential impact. We aim to promote methodological integrity and the responsible interpretation of epidemiological data in public health research, ensuring that findings contribute constructively to the scientific and policy landscape. The main methodological concerns are summarized in Table 1.

**Table 1:** Key points, authors' assertions, and proposed refinements.

**Key point**	**Author's statement (2)**	**Authors' assertions (2)**	**Critical refinements**
Selection bias	“This study focused on the most serious cases of COVID-19.”	Severe SARS cases represent the most critical population.	The study limits generalizability to broader COVID-19 populations by focusing solely on severe SARS cases. These findings contrast with more comprehensive analyses by Ranzani et al. (7) and Castro et al. (8), which evaluate all hospitalized COVID-19 patients and regional disparities.
Exclusion of early deaths	“Only individuals with at least a 3-month interval between first symptoms and death were included.”	Patients dying early were excluded.	Excluding deaths within 3 months omits the most vulnerable patients, introducing survival bias. This underestimates mortality in resource-constrained regions, particularly in the North, as shown by Castro et al. (8) and Silva et al. (9). Including early deaths would provide a fuller mortality profile.
Missing data	“The completeness of data for non-mandatory fields generates a significant loss of information.”	20%−31% missing data for comorbidities.	Missing data on critical predictors of mortality undermines reliability. Specifically, diabetes (29%), kidney disease (31%), and heart disease (18%) were marked as “unknown.” These gaps, without imputation or sensitivity analyses, compromise the validity of conclusions regarding comorbidities.
Database limitations	“SIVEP-Gripe^*^ was used to track SARS cases.”	The database was designed for short-term surveillance	SIVEP-Gripe^*^ is primarily structured for acute event monitoring, focusing on the notification and closure of severe acute respiratory syndrome (SARS) cases. Its lack of a longitudinal structure limits its capacity to capture post-hospitalization outcomes or long-term mortality. Integrating complementary databases, such as the Mortality Information System (SIM), enhances data reliability, reduces misclassification, and ensures accurate long-term analyses.
Vaccination impact	“The risk of death was reduced in the medium term but doubled in the long term for vaccinated individuals.”	Suggests a reversal of vaccine benefits.	The study suggests a reversal of vaccine benefits. However, this claim lacks adjustments for critical factors, including vaccine type (e.g., mRNA, adenovirus, inactivated virus), dose intervals, and patient frailty. While vaccines reduce early mortality among vulnerable populations, this protective effect is underexplored. The absence of stratified analyses and comparisons with large-scale studies (7) limits the understanding of differential vaccine performance. Proper adjustments are crucial to separate the effects of vaccination from underlying vulnerabilities and regional disparities.
Regional mortality	“The North and Northeast regions showed the highest mortality.”	Mortality in the North and Northeast reflects systemic inequities and lower vaccination coverage compared to other regions.	The findings align with robust evidence from Castro et al. (8) and Silva et al. (9), attributing higher mortality in the North and Northeast to systemic healthcare inequities, ICU shortages, and reliance on the public sector. These regional disparities and lower vaccination rates may have influenced the study's conclusions. Proper adjustments are essential to account for these imbalances and avoid misattributing mortality patterns to vaccination.
Statistical models	“Classic Cox, Cox mixed-effects, and Cox frailty models were used.”	Three models analyzed mortality risk.	The absence of diagnostic tests (e.g., Schoenfeld residuals) raises concerns about the validity of proportional hazard assumptions. Multilevel Cox models incorporating regional covariates and considering competing risks would enhance the statistical rigor of the analysis.

This table summarizes the main issues identified in the original study, the authors' statements regarding these points, and suggested refinements to address methodological limitations and improve the validity of the findings ^*^SIVEP -Epidemiological Surveillance Information System for Influenza (Sistema de Informação de Vigilância Epidemiológica da Gripe in Portuguese).

## Discussion

The methodological approach adopted by Rodrigues and Andrade (2) presents critical limitations, particularly the exclusion of early deaths, which introduces substantial survival bias and affects the statistical modeling of post-COVID-19 mortality. By omitting patients who succumbed early, the study inherently underestimates overall mortality, especially in resource-limited regions, skewing its long-term conclusions.

Another fundamental concern is the lack of verification of the proportional hazards assumption in their Cox regression model. This assumption is central to the validity of the hazard ratios (HRs) reported, as it dictates that the relationship between each covariate (e.g., vaccination status, age, and comorbidities) and mortality remains constant over time. Failure to assess this assumption, particularly through Schoenfeld residual analysis (3), raises serious concerns about the reliability of their findings.

Additionally, the reliance on the SIVEP-Gripe database represents a critical methodological constraint, as recently highlighted by the Brazilian Ministry of Health (1). This database was developed specifically for acute surveillance of severe respiratory cases and was not designed or validated for tracking long-term mortality post-hospitalization. Integrating SIVEP-Gripe data with more comprehensive databases, such as the Mortality Information System (SIM), would significantly improve mortality assessments and reduce the risks of misclassification and underreporting.

The authors report an increased long-term mortality risk among vaccinated individuals, a finding that contradicts extensive population-based studies demonstrating vaccine effectiveness in reducing mortality (4, 5). A plausible explanation for this discrepancy lies in the potential violation of the proportional hazards assumption. The protective effect of vaccination is strongest in the early months following infection but may appear to diminish over time due to the selection bias inherent in vaccination prioritization—i.e., more vulnerable individuals, who already have a higher long-term mortality risk, were vaccinated earlier (2, 6). If this time-dependent effect was not accounted for, the study may be misinterpreting an expected epidemiological trend as a causal link between vaccination and increased mortality.

A standard statistical approach to verify this issue would be to calculate Schoenfeld residuals for key covariates, particularly vaccination status, and examine whether the assumption of proportionality holds (3). If these residuals exhibited a systematic trend over time rather than random dispersion, it would indicate a violation of the proportional hazards assumption, necessitating a more flexible modeling strategy, such as time-dependent Cox models or stratified analyses (6). The omission of this verification is a serious methodological flaw, as it leaves open the possibility that the reported increase in long-term mortality among vaccinated individuals is a statistical artifact rather than a genuine epidemiological effect.

Furthermore, the study fails to adequately consider vaccination's impact beyond mortality, including hospitalization duration, reinfection rates, and morbidity outcomes. A more comprehensive evaluation of vaccine efficacy requires assessing these broader outcomes, particularly in populations with high frailty and preexisting conditions, where confounding factors must be carefully addressed to avoid misinterpretation (4, 5).

The methodological concerns raised by this study have also been acknowledged at an institutional level. The Brazilian Ministry of Health (1) issued a formal statement in the official website emphasizing that the SIVEP-Gripe database was not designed for long-term mortality analysis and that misinterpretation of its data could lead to misleading conclusions about vaccine effectiveness. The Ministry reaffirmed that vaccines remain a crucial tool in reducing COVID-19 mortality and preventing severe disease, stressing that the study's findings should not be used to undermine vaccination campaigns.

In conclusion, failure to incorporate these necessary methodological safeguards compromises the validity of the study's conclusions. Without proper adjustments for time-dependent effects and verification of statistical assumptions, the reported associations risk being misinterpreted, potentially disseminating misinformation, misguiding public health policies, and reinforcing misleading narratives regarding vaccine efficacy and safety.

## Author's note

Dr. Rubens C. Costa-Filho is a PhD affiliated with the Laboratory of Immunopharmacology, IOC/FIOCRUZ, and the Estácio Medical School-IDOMED, where Dr. Rubens C. Costa-Filho currently serves as a Professor of Pharmacology. Dr. José Paulo G. Leite is a PhD and Senior Researcher at the Laboratory of Comparative and Environmental Virology, IOC/FIOCRUZ, Rio de Janeiro. Dr. Marco Aurélio Horta holds a PhD and is affiliated with the Laboratory of Hantavirus and Rickettsial Disease, IOC/FIOCRUZ, Rio de Janeiro. Dr. Felipe Gomes Naveca is a PhD affiliated with the Oswaldo Cruz Institute, Fiocruz, Rio de Janeiro, and the Leônidas and Maria Deane Institute, Fiocruz, Amazonas. Dr. Felipe Saddy holds a PhD and serves as the Coordinator of the Intensive Care Unit (ICU) at Hospital Pró-Cardíaco in Rio de Janeiro, Brazil. Dr. Hugo Caire Castro-Faria-Neto holds a PhD and is a Senior Researcher at the Laboratory of Immunopharmacology, IOC/FIOCRUZ, and also a Professor of Pharmacology at Estácio Medical School – IDOMED in Rio de Janeiro, Brazil.
